# Antibiotic Susceptibility Testing (AST) Reports: A Basis for Environmental/Epidemiological Surveillance and Infection Control Amongst Environmental *Vibrio cholerae*

**DOI:** 10.3390/ijerph17165685

**Published:** 2020-08-06

**Authors:** Bright E. Igere, Anthony I. Okoh, Uchechukwu U. Nwodo

**Affiliations:** 1 Applied and Environmental Microbiology Research Group, Department of Biochemistry and Microbiology, University of Fort Hare, Alice 5700, South Africa; okoh@ufh.ac.za (A.I.O.); unwodo@ufh.ac.za (U.U.N.); 2 SAMRC Microbial Water Quality Monitoring Centre, University of Fort Hare, Alice 5700, South Africa

**Keywords:** antibiotic susceptibility testing (AST), *Vibrio cholerae*, epidemiology, multiple antibiotic resistant index MARI

## Abstract

Distribution, investigation, surveillance and control (DISC) of cholera outbreaks in endemic/non-endemic regions has been a concerted approach towards the management of the causal pathogen. Relevant organization, government, health systems and the public have implemented several steps towards controlling the menace, yet pathogen continues to occur with diverse phenotypes/genotypes of high clinical and epidemiological relevance. The study determines antibiotic susceptibility/resistance pattern of *Vibrio cholerae* isolates retrieved from six domestic water sources between March and August 2018. Serological and molecular typing methods (polymerase chain reaction or PCR) were used to confirm the isolates identity. Antibiotic susceptibility testing was conducted using six commonly employed antibiotics of *V. cholerae* according to the recommendation of Clinical Laboratory Standard and European Committee for Antimicrobial Susceptibility Testing with other relevant antibiotics of investigative epidemiology and infection control, employing both disc diffusion test and PCR gene detection. Samples presumptive counts ranged between 1.10 to 7.91 log10 CFU/mL. Amongst the 759 presumptive isolates retrieved, sixty-one were confirmed as *V. cholerae* which were further serogrouped as Non-O1/Non-O139 *V. cholerae*. Various *V. cholerae* resistant phenotypes/genoytypes were detected vis: carbapenemase (CR-Vc; 31.1%/5.3%). New Delhi Metallobetalactamase (NDM-1-Vc; 23.0%/42.5%), extended spectrum betalactamase (ESBL-Vc; 42.6%/blaTEM:86,7%), chloramphenicol resistance (62.3%/Flor: 46.2%}, tetracycline resistance (70.5%/46.7%), AmpC resistance (21.0 (34.4%/56.7%)) and various other resistant genotypes/phenotypes. It was observed that more than 50% of the confirmed *V. cholerae* isolates possess resistance to two or more antibiotic classes/groups with multiple antibiotic resistance index (MARI) ranging from 0.031 to 0.5. This observation provides necessary information and updates for surveillance, planning and implementation of control strategies for cholera. It would also encourage decision making, formulation of policy by the government and cholera control authorities.

## 1. Introduction

Diseases associated with *Vibrio cholerae*, its patho-significance as well as clinical relevance continues to emerge and re-emerge both globally and various endemic/non-endemic regions of Africa. *V. cholerae* is a Gram-negative bacterium belonging to the genus *Vibrio* that thrives mainly in estuarine and marine environments as free living bacteria and a colonizer of diverse milieu [1,2,3]. The reports that depict the potential pathogen as a resident flora of estuaries and coastal waters have clear indications that it is a foe-like ally or an adversary-like friend which thrives in the environment with man, animals, plant, and other living organisms. Such co-existence in the estuaries allows the pathogen to experience both the harsh and favourable environmental changes, the chemical nature (physicochemical indices) of the environment, the activities of the environment both take-in and out including the hydrophobicity and hydrophilicity. According to Crowell [4], these characteristics which are elicited based on environmental changes are inherent. Various studies have asserted that such changes in the environment are associated with indiscriminate release of diverse wastes. In the study by Manaia et al. [5], it was reported that bulk of all living species release their waste into water bodies, where bacteria in the water bodies as well as those carried over bacteria (industrial, human, animal and plant origin) thrives. The bacteria proliferate, utilizing the rich water nutrients and chemical agent from effluents release to develop resistant genes. Other studies also added that antibiotic resistance resulted as survival mechanism of various Gram-negative pathogens due to inappropriate usage and use of antibiotics as therapeutic prophylactics against pathogens [6,7]. The organisms in such an environment thereafter muster survival strategies, using developed immunity [8,9,10], multiple antibiotic resistance (MAR) [11,12,13,14,15,16], multiple patho-genetically diverse genes [4,17,18,19,20], multiple biochemical pathways [21,22,23,24,25,26,27] and multiple epidemiological (pandemic/epidemic) variants virulent determinants [18,19]. In the environment, various antimicrobial/chemical agents have been released as waste and/or applied either to kill or reduce the quantity of bacterial (as bacteriocides) in both terrestrial and aquaculture [28] which have also encouraged development of multiple antibiotic resistance (MAR) amongst halophilic pathogens such as *V. cholerae* [29]. Such antibiotic resistances amongst environmental bacterial specie pose threats to both humans and the environment as there is possibility of transfer or sharing of multiple drug resistant genes amongst potential pathogens via horizontal gene transfer [6]. Other recent studies conducted by various investigators have affirmed that *V. cholerae* thrive in the environment, remodel its surface lipopolysaccharide (LPS) and protect itself from host defenses, drug agents and immune scrutiny [15]. One surface enzyme expressed by members of *V. cholerae* with such character is the AlmG which plays a pivotal role in peptide-bound antibiotic resistance and survival against host defense [13,15]. Numerous studies have associated resistance to peptide-bound antibiotics with phosphoethanolamine modification of lipid A [13] which is common amongst the El Tor *V. cholerae* strains. Various Gram-negative bacteria investigations have reported high protease resistance or peptide-bound resistance prevalence including the *ESβLs, CMY, NDM1, MOX, DHA, FOX and AmpC β*–lactamases genes [30,31,32,33]. Such antibiotic hydrolyzing enzymes are reported to be prevalent amongst most Gram-negative pathogens and these resistances are spreading among them by conjugative plasmid transfer [21,34,35,36].

Over the years, one revealing tool for in vitro determination of susceptibility/resistant dynamics has been antibiotic susceptibility testing (AST). AST is conducted on potential pathogens which have penchant to proliferate in the environment, implicated in broad range (epidemic/pandemic) infection cases and there is reported resistance and/or multiple antibiotic resistance to commonly employed antibiotics. This implies that AST must be applied with appropriateness on pathogens while considering management/control of disease cases as previously suggested by EUCAST/CLSI [37,38]. Bacteria have been reported to acquire antibiotic resistance since the discovery of antibiotics which pose super-challenge for antibiotic choice when considering therapeutic management. Such acquisition may result resistant phenotype or genotypes amongst potential pathogens, as AST remains the in vitro determinative technique employed. In addition, the detection/visualization of such molecular based resistant genotypes involve using agarose gel electrophoresis with standardized Polymerase chain reaction (PCR) technique as applied in various studies [6,36]. The successful thriving of *V. cholerae* in the water environment may expose the potential pathogen to the problem of failure to control as the potential pathogen may have acquired multiple antibiotic resistant genes from the environment [29]. Such attribute of *V. cholerae* especially the environmental strains amongst safe water deprived Municipalities remains a concern, as unhealthy water release may encourage spread/acquisition of resistance and genomic island [12,39,40,41,42,43]. It is worthy of note that early detection of such emerging resistant determinants in the environment (and/or potential pathogens) be investigated since it serves as a guide towards policy planning, decision making, infection control, epidemiology and surveillance [44]. It is to this end this study intends to access antibiotic susceptibility as a basic tool for epidemiological surveillance and infection control amongst environmental *V. cholerae.*

## 2. Materials and Methods

### 2.1. Study Area

The study was conducted within three local Mmunicipalities (Amahlathi, Chris Hani and Lukhanji local municipalities), which are located at 32°38.381′ S, 026°56.165′ E and 31°54.548′ S, 026°50.715′ E within two district municipalities (Amhathole and Chris Hani district municipalities). Final effluents from three wastewater treatment units (WWTPs), receiving water bodies (RWS), rivers (R), earth canals (IC), dams (D) and tap water (T) were sampled from these municipalities. These treatment plants receive municipal wastewater from household and domestic usage. Rivers, dams and earth canal water are used for animal husbandary, irrigation of farmlands, recreational activities, household/domestic activities and drinking in rare situations. Most irrigated farmlands (both local and commercial farmlands) cultivate and harvest large variety of vegetables which are commercialized and used as food in both sub-urban and peri-urban areas of the study municipalities.

### 2.2. Sample Collection

Water samples were collected from six domestic water sources between March and August 2018. Some of the sampled wastewater treatment plants were non-functional during the period of study yet still release water into the environment, which at the receiving communities, is used for both irrigation, domestic and/or outdoor activities. One thousand and eighty water samples were collected using sterile one L screw-capped Nalgene glass bottles and transported to the Applied and Environmental Microbiology Research Group (AEMREG) advanced research laboratory of the University of Fort Hare (Alice, South Africa) in cooler boxes filled with ice packs for analysis. Forty-five samples were processed every month for each sample site as follows WWTP/RWS: 20, R: 10, D: 5, IC: 5, T: 5. Bottles were pre-cleaned by washing with non-ionic detergent and rinsed in running tap water. A further pre-cleaning to avoid cross contamination includes autoclave, treating with fifty percent hydrochloric acid (HCl-50%), rinsing with sterile deionised water, addition of 1.7 mL sterile one percent sodium thiosulphate solution aseptically before collecting samples at various sampling points and samples analysed within 8 h of collection.

### 2.3. Presumptive Vibrio Cholerae Numerical Density or Count

With few modifications, the methods previously described by Huq et al. [45] and Uddin et al. [46] were applied. Water samples were filtered using a vacuum pump/pressure pump model No.DOA-P730-BN (Life Science PALL, Gauteng, Pretoria, South Africa) and standard membrane filtration techniques after a 10-fold dilution using 0.45 µm nitrocellulose filter paper (Merck KGaA, Darmstadt, Germany). A triplicate filter membrane was plated onto pre-prepared thiosulphate citrate bile salts-sucrose (TCBS) agar and incubated at 37 °C for 24 h [47,48]. Presumptive *V. cholerae* triplicate count was expressed in colony forming units per millilitres (CFU/mL) of water for both yellow and green colonies. Colonial morphology and cultural characteristic of representative colony were observed as suspected colonies. Isolates were subcultured onto TCBS and subsequently onto nutrient agar to ascertain the purity. Five to ten suspected colonies per-plate were randomly picked for each sample and stored in aliquot of glycerol stock.

### 2.4. Presumptive Identification and Biochemical Reaction

Pure *V. cholerae* isolates were tested using Gram reaction, oxidase reaction, motility, Voges Proskauer test (VP-test) and D-mannitol catabolism. An invitro presumptive biochemical and virulence indices of the isolates were determined using the string test, protease production, lipase production, lecithinase production and cholera red production. Although these tests were not sufficient for a specific identity, it is used as a preliminary/presumptive pathotyping protocol especially for the *V. cholerae* members. Isolates that show positive and/or negative reaction to these multiple tests were presumptively selected for confirmation as *V. cholerae* or non-*Vibrio* members.

#### 2.4.1. Serological Identification and Molecular (PCR) Confirmation of Isolates

The MAS-AGGL-M11003 *V. cholerae* Inaba antisera (411900) and MAS-AGGL-M11004 *V. cholerae* Ogawa antisera (411901) were purchased from Davis Diagnostics (Pty) Ltd., Randburg Gauteng, South Africa) [49]. Following manufacturer’s instructions, serological test was conducted using a single pure colony of 24 h old culture. Other serological methods employed the use of target specific primer pairs for sero-grouping of pathogens see Table 1 for details.

#### 2.4.2. Extraction of Genomic DNA

Genomic DNA of *V. cholerae* was extracted following the boiling method previously described by Maugeri et al. [52] with a few modifications. An overnight nutrient broth culture of each isolate aliquot were subcultured into a sterile 1.5 mL microfuge tube, centrifuged for 2 min at high speed to pellet cells and washed twice with phosphate buffered saline. Cell pellets were then suspended in 500 µL sterile distilled water or deionised water, boiled at 100 °C for 10 min in a pre-heated heating block (Techne heating block Dri-Block, DB-3D; Gauteng, Pretoria, South Africa). The heated suspension is centrifuged for 5 min at 13.500 rpm and supernatant is collected. The collected supernatant is then stored at −20 °C until used as DNA template for PCR.

#### 2.4.3. Target-Specific Identification of *V. Cholerae* Using PCR and Agarose Gel Electrophoresis

Target specific primers sequences were retrieved from previous investigators reports and sent to Inqaba Biotechnical industries (Pty) Ltd. (Hatfield Pretoria, South Africa) for synthesis and reports. The genus specific primer sequence (specific *16SrRNA* primer sequences) and specie specific *OmpW* gene (304bp) were used as described in Table 1 above. Confirmed *V. cholerae* isolates were further accessed for other resistant phenotype, genotype, sero-group and biotype. PCR (T100^TM^ thermal cycler, Bio-Rad, Hercules, CA, USA) and cycling conditions were conducted using a 200 µL microfuge tube. Approximately 50 picomolar to 1 μM DNA extract was used in a PCR tube with a final volume of 25 µL, a GoTaq ^℗^G2 green master mix supplied in 2× Green GoTaq^℗^G2 reaction buffer containing pH: 8.5, dNTPs {400 µM each of dATP, dGTP, dCTP and dTTP}, 3 mM MgCl_2_ and GoTaq^℗^G2 DNA polymerase at optimal concentration for efficient PCR amplification as specified by Promega Corporation (Madison, WI, USA; www.promega.com) were used. Primer concentration of 0.5 µM, thermocycling condition for 16SrRNA gene were 4 min at 94.0 °C followed by 35 cycles of 94.0 °C for 1 min, 53.0 °C for 1 min and 72.0 °C for 1minute and a final extension step at 72.0 °C for 8 min, while that of OmpW gene was 3 min at 94.0 °C followed by 35 cycles of 93.0 °C for 45 s, 64.0 °C for 1 min and 72.0 °C for 2 min and a final extension step at 72 0 °C for 8 min Other resistance gene cycling conditions are reported in Table 1. Agarose electrophoresis was carried out using a Sigma-based tris acetate-EDTA (TAE) of 50× (Sigma Aldrich, Dorset, UK) which is re-constituted to a 1× TAE running buffer. Gel was prepared by weighing 1.5 g of agarose powder (Sigma Aldrich), dissolved in 100 mL of running buffer and heated to boiling. The prepared gel is casted on a minigel tray (Anachem, Dorset, UK), allowed to polymerise, placed carefully in an electrophoresis tank filled with 1× TAE Buffer and electrophoresed (electrophoresis machine CLS-AG100, Warwickshire, UK) at 100 V for 50 min. The gel was visualized on a Gel doc imaging system (Bio Rad Hercules, CA, USA).

### 2.5. Inoculum Preparation and Antibiotic Susceptibility Testing (AST) of V. Cholerae

The disc diffusion method previously described by Kirby-Bauer was employed for Antibiotic Susceptibility testing (AST) following the European Committee for Antimicrobial Susceptibility Testing (EUCAST) and Clinical and Laboratory Standards Institute (CLSI) [37,38,46,53] documented criteria for AST determination with minimal modifications. An overnight culture of test organism was directly suspended in 5 mL aliquot of pre-prepared sterile normal saline in pre-cleaned and sterile test tubes. Test organism suspension was made to an inoculum density equivalent to 0.5 McFarland standards or 10^6^ CFU/mL. Using sterile cotton-wool swab sticks, each test suspension was streaked onto Mueller-Hinton agar plates (MHAP). Thirty two antibiotic discs were commercially obtained from Mast Diagnostics (Merseyside, UK) via Davies Diagnostics (Pty) Ltd. and aseptically placed on a streaked MH agar plates. The thirty-two antibiotic discs used were cephalosporins or cephem: (ceftazidime (CAZ-30 µg)), (cefepime (CPM-30 µg)), (cefotaxime (CTX-30 µg)), (ceftriaxone (CRO-30 µg)), (cefuroxime (CXM-30 µg)), (cephalexin (CFX-30 µg)), (cephalothin (KF-30 µg)), (cefazolin (CZ-30 µg)), citrofurans: (nitrofurantoin (NI-200 μg)), phenicols: (chloramphenicol (C-30 μg)), folate pathway inhibitor: (trimethoprime-sulfamethoxazole (TS-25 μg)), penicillins: (ampicillin (Amp-10 μg)), β-lactam/β-lactamase inhibitors: (amoxicillin-clavulanate (AUG-30 μg)), piperacillin-tazobactam (PTZ-110 μg)), (ampicillin-sulbactam (SAM-20 μg)), aminoglycosides: (gentamycin (Gm-30 μg)), amikacin (AK-30 μg)), (streptomycin (S-30 μg)), (kanamycin (K-30 μg)), carbapenems: (imipenem (Imi-30 μg)), (ertapenem (ETP-10 μg)), (meropenem (Mem-10 μg)), (doripenem (Dor-10 μg)), tetracyclines: (tetracycline (T-30 μg)), (doxycycline (DXT-30 μg)), (oxytetracycline (OT-30 µg)), macrolides: (erythromycin (E-15 µg,)), (azithromycin (ATH-15 μg)), fluoroquinolones: {ciprofloxacin (CIP-5 µg)}, (levofloxacin (Lev-5 µg)), (nalidixic acid (NA-30 µg)), (norfloxacin (Nor-10 µg)) and lipopeptides: (polymyxin B (PB-300 µg)). Clear inhibition zones were measured in millimeter diameters using meter rule and interpreted by applying the EUCAST and CLSI guidelines [7,37,53]. The inoculated agar plates were allowed to stand for 10 min and incubated at 37 °C for 18–24 h. Measurements were recorded and interpreted as either resistant (R), intermediate (I) and/or sensitive (S) according to the EUCAST and CLSI guidelines [37,46,53].

#### 2.5.1. Multiple Antibiotic Resistance Index (MARI) Determination and Statistical Analysis

Multiple antibiotic resistance index (MARI) determination was applied using the method of Odjadjare et al. [7] to determine the resistant nature of pathogens collected during the study. This was done using the formula: MARI = a/b, where “a” is the number of antibiotics to which resistance was observed amongst isolates while “b” is the total number of antibiotics used during study. ANOVA tool was employed on the various presumptive *V. cholerae* mean cell density/count which was obtained from the various water samples, using pearsons correlation to determine level of significances in counts/density of samples analysed in relation to sites. While the PAleontological Statistics Version 3.14 (the past3. software package 3.14 Oslo, Norway) [54] was used on other divergent and cluster studies. The absence/presence of resistance amongst isolates after EUCAST/CLSI interpretative guidelines indicates strain divergence or similarity. A dendrogram was created by neighbour-joining (NJ) [54,55] using Euclidean similarity index of the past3.zip software package 3.14 and an Excel spreadsheet were also used to produce other tables and figures.

#### 2.5.2. Phenotypic Detection of AmpC Resistance

Isolates that were resistant to cefazolin, cephalothin, cephalexin, ceftriaxone and cefotaxime in the previous invitro AST determination were selected for AmpC analysis. The disc approximation test (DAT) method was employed as previously described by Gupta et al. [31]. A ceftazidime disk (CAZ-30 µg) was placed at the center of a freshly inoculated MHA plate, then imipenem (IMI-10 µg), cephalothin (KF-30 µg) or cefazolin (CZ-30 µg) and amoxicillin-clavulanate (AUG-20/10 µg) disks were placed at a 20 mm each, away from ceftazidime disk and incubate 18–24 h at 35 °C. The observation of obvious blunting or flattening of the zone of inhibition between ceftazidime disk and other inducing antibiotics disks (imipenem, cefoxitin and amoxicillin-clavulanate) indicates a positive result for *Amp*C production. The AmpC disk test and boronic acid disk test method (BADT) were also employed with few modification and organisms that showed an increase in zone of inhibition of ≥5 is indicated as AmpC positive detection [31,32].

#### 2.5.3. Phenotypic Detection of ESβL

Isolates that were resistant to ceftazidime disk (CAZ-30 µg), cefotaxime CTX-30 µg) and ceftriazone CRO-30 following the interpretation by CLSI [37,46,53] guidelines were considered as potential ESβL producers. The combined disc (CDST) and double disc synergy test (DDST) method were employed. Commercially available ESβL detection antibiotics were purchased from Mast Diagnostics via Davies Diagnostics (Pty) Ltd. 141 (Oak Avenue, Ferndale, Randburg, 2194, Gauteng, South Africa) and aseptically applied to streaked MH agar plates.

##### The Double Disc Synergy Test (DDST)

The DDST was conducted on pre-prepared MHA plates with discs containing cefotaxime (30 µg) and piperacillin/tazobactam (100 µg/10 µg) respectively, placed 20 mm apart (centre to centre). An extension or protrusion of the inhibition zone around the cefotaxime disc towards the piperacillin/tazobactam disc is indicative for ESBL production. *Escherichia coli* strains ATCC 25922 was used as quality control strains for the DDST [34,35,51].

##### The Combined Disc Synergy Test (CDST)

The CDST was further used to confirm ESβL producing isolates following manufacturer’s instructions using ceftazidime (30 µg), ceftazidime/clavulanic acid (30/10 µg). An observation of ≥5 mm increase in zone of inhibition diameter for ceftazidime in the synergy test when compared with the ceftazidime alone confirms production of ESβL by isolate.

#### 2.5.4. Phenotypic Detection of Carbapenem Resistance

Isolates that were resistant to ertapenem (ETP-10) and some members of the cephalosporins such as cefotaxime (CTX-30), ceftazidime (CAZ-30) and ceftriaxone (CRO-30) were suspected for carbapenemase production. The Modified Hodge Test (MHT) method and the EDTA-Ertapenem synergy test were applied. The EDTA-Disk Diffusion Synergy Test is done as follows; Briefly a 0.5 McFarland standardized saline suspension dilution of both test organism and control strain (*E. coli* ATCC 25922) in appropriately labeled tubes were prepared. Standardized suspensions were inoculated onto freshly prepared Mueller Hinton agar plates (MHAP) and allowed to stand for 10 munites. Two disc each of ertapenem and imipenem were placed 20 mm apart (centre to centre) on the surface of solid agar plates and 10 µL of pre-prepared 0.5M EDTA was added to one of the disc while the other was not enhanced. Plates containing preparation were incubated for 18–24 h. The observation of ≥4 mm zone of inhibition in the EDTA fortified disc is indicative of a carbapenemase producing isolate. The control strains were treated in similar procedure as the test organisms. Alternatively, imipenem (IMI-10 µg) disc may be used by placing a 6 mm in diameter Whatman filter paper no. 2 at 10 mm apart from the edge of Imipenem disc. A 10 µL solution of 0.5 M EDTA is then added to disc (approximately 1.5 mg/disc) and incubated overnight. The observation of an enlarged clear inhibition zone is indicative for positive EDTA synergy test [37,46,51].

#### 2.5.5. MHT Confirmation

The method was confirmed by centrally placing meropenem (MEM-10) disc on the surface of previously inoculated MHAP-containing control strain (*E. coli* ATCC 25922). The standardized test isolates were then inoculated by streaking a straight line lawn of the test organism from the antibiotics down towards the edge of the plate. The streaked lawn of test organism was then allowed to stand for 3–5 min and preparation was incubated for 18–24 h at 35 °C ± 2 °C in ambient air. After 18–24 h of incubation, plates were examined for any clover leaf-type indentations at culture intersect of both test organism and E. coli 25922, around the carbapenem zone of inhibition. A positive test is reported for the observation of clover leaf-like indentation of E. coli 25922, growth along test organism growth streak within disk diffusion zone, while negative test is reported on observing no growth of control strain (E. coli 25922) around the test organism growth streak since the antibiotic inhibited the growth of organism. These described methods were used to confirm phenotypic Detection of New Delhi Metallo-β-lactamase-1 (NDM-1 gene) [37,51].

## 3. Results

### 3.1. Genera Specific 16SrRNA PCR Gene Detection

The study retrieved 759 presumptive Vibrio isolates from 1080 samples, which were collected from all sampled water sources as shown in Table 2 as follows; H; {WWTP/RWS: 0, R: 57, D: 34, IC: 34} C; {WWTP/RWS: 17, R: 69, D: 58, IC: 44}, Q; {WWTP/RWS: 95, R: 73, D: 59, IC: 52}, CF; {WWTP/RWS: 46, R: 49, D: 38, IC: 34}. Amongst the 759 presumptive Vibrio isolates retrieved from the study, seven hundred and forty-two were positive to the 16SrRNA gene detection confirming 97.8% (742/759) as Vibrio species (Supplementary Figure SI 1 and Table 2).

### 3.2. V. Cholerae Cell Density

The numerical/population density of *V. cholerae* from various water sources, mean cell counts and standard error per sampling months are as shown in the Table 2 and Table 3. Higher plate count/density was recorded in the first two months of sampling with an observed increase/decrease in presumptive *Vibrio* counts. This indicates that there is inappropriateness or a possible compromise in the standard of released effluent hence an undulating *Vibrio*-density in the assessed release. This is also evident in the wastewater treatment plants (WWTPs) as the mean count in QT WWTP was higher amongst the studied WWTP in the study municipalities (Table 3) This undulating density has also influenced the numerical-density of presumptive *Vibrio* counts in the various water sampled (R, D, IC) since the release flows into the various water sources as shown in Table 2. Although, presumptive enumeration revealed higher population of presumptive *Vibrio* species in the environment, the PCR detection confirmed 61/759 (8.04%) *V. cholerae* in the studied environment with higher population observed in wastewater final effluent/receiving water shed and rivers amongst sample site Q as depicted in the Table 2 below.

### 3.3. Antibiotic Susceptibility Test (AST) and Profile of V. Cholerae Isolates

**Figure 1:**
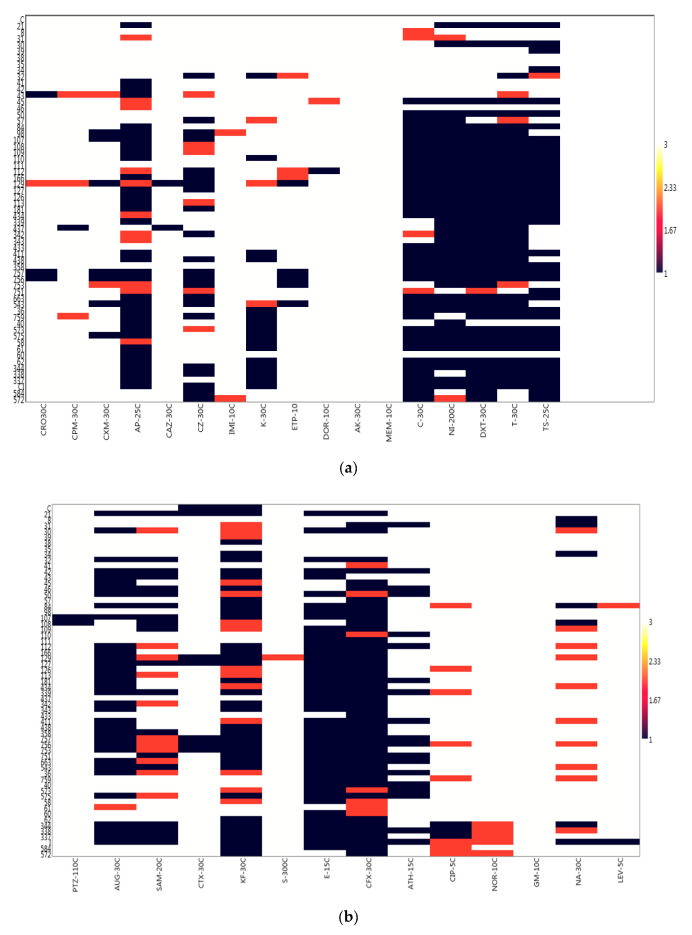
(**a**) The antibiogram of the various *V. cholerae* isolates to relevant antibiotics. (**b**) The antibiogram of isolates to other antibiotics use for Gram negative or Enterobacteriaceae members.

The study accessed the antibiogram of the confirmed *V. cholerae* isolates collected from environmental and domestic water sources using both oral and parenteral antibiotics as specified in CLSI [37] and other antibiotics as suggested in EUCAST [38] guidelines. The antibiogram and profile of the *V. cholerae* isolates (Table 4) revealed that all the isolates 61/61 (100%) were sensitive to gentamicin (GM-10), meropenem (Mem-10) and amikacin (AK-30) while isolates numbers ranging from 50 to 60 (82.0–98.4%) were sensitive to ciprofloxacin CIP-5, cefuroxime CXM-30, ertapenem ETP-10, cefotaxime CTX-30, norfloxacin NOR-10, ceftriaxone CRO-30, cefepime CPM-30, ceftazidime CAZ-30, imipenem IMI-10, doripenem DOR-10, piperacillin-tazobactam PTZ-110, levofloxacin LEV-5 and streptomycin S-300. Resistance was observed in 77.1% of isolates to an oral cephem, cephalexin CFX-30 (47/61, 77.1%), erythromycin E-15 (46/61, 75.4%), doxycycline DXT-30 (45/61, 73.8%), chloramphenicol C-30 (39/61, 63.9%), tetracycline T-30 (43/61, 70.5%), trimethoprime-sulfamethoxazole TS-25 (40/61, 65.6%) as shown in the antibiotic susceptibility profile (Figure 1a,b and Table 5 below). It was also revealed from the study that some of the isolates were resistant to important antibiotics of *V. cholerae*, non-relevant antibiotics and other antibiotics of epidemiological relevance as shown in Table 5. Such resistant phenotypes are important biomarkers that may pose threat to the management of cholera in any outbreak. According to CLSI [37,39,46] ampicillin, azithromycin, chloramphenicol, tetracycline, doxycycline and trimethoprime-sulfamethoxazole are very important antibiotics for the *V. cholerae* management/control of cholera cases. The study reveal high level of resistance to these members of antibiotics (see Table 4) except azithromycin which is a pointer to the therapeutic failure observed and reported by various investigators in the control/management of the potential pathogen using those antibiotics.

The above is a matrix plot of the past3 statistical software 3.14 Version (Oslo, Norway) [54], it was use with the generated code for numbers ranging from 1 to 3 where 1 represents resistance to particular antibiotic, 2 represents an intermediate susceptibility result and 3 represents high susceptibility as interpreted from the EUCAST/CLSI [38,53]. The various colour reads in the keys: Resistance (Deep blue), intermediate (pink) and sensitive (White). It shows that antibiotic pressure or resistance is higher amongst the commonly applied antibiotics. (b) The above is a matrix plot of the past3. statistical software 3.14 Version, it was use with the generated code for numbers ranging from 1 to 3 where 1 represents resistance to a particular antibiotic, 2 represents an intermediate susceptibility result and 3 represents high susceptibility as interpreted from the CLSI [53]. The various colour reads in the keys: Resistance (Deep blue), intermediate (pink) and sensitive (White). It shows that antibiotic pressure or resistance is higher amongst the β-lactam/inhibitor antibiotic members.

#### 3.3.1. Occurrence of AmpC Resistance

**Figure 2:**
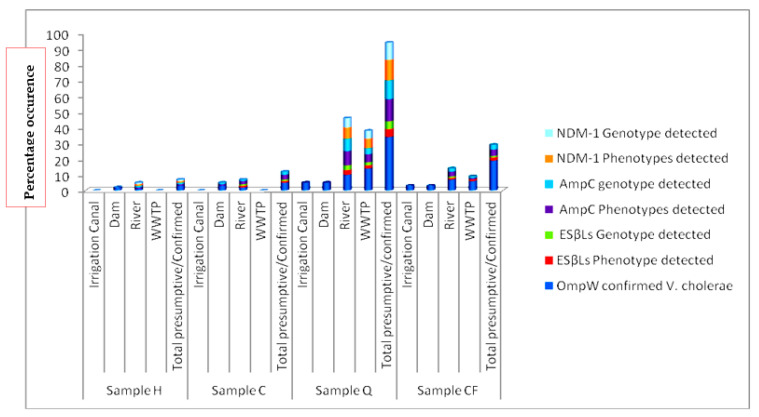
Distribution of various resistant phenotypes and genotypes in the various sampling sites for all nonO1/nonO139 *V. cholerae* strains retrieved during the study.

Table 6, Figure 2 and Supplementary Figure SI 8 shows the percentage occurrence of *AmpC* resistant *V. cholerae* in the environmental water sampled during the study. It reveals that, present in the receiving river body of the study area are high distribution of class C resistant *β-lactamase* phenotype. It shows that 21/61 (34.4%) of the isolated *V. cholerae* were observed to express *AmpC* resistance.

#### 3.3.2. Occurrence of ESβLs Resistance

Consistent in the antibiogram (see Table 5 and Table 6) from the study is the occurrence of resistance to members of the third-generation cephalosporin antibiotic groups. It was observed that 26/61 (42.6%) of the isolated *V. cholerae* were shown to produce *ESβL*. The distribution of *ESβL* producing *V. cholerae* phenotypes is summarized in Table 6, Figure 2, Supplementary Figures SI 9 and SI 10 for selected positive isolates. The pattern observed for the PCR gene detection of the resistant genes was also shown in Supplementary Figure SI 3.

#### 3.3.3. Occurrence of NDM-1 Resistance

The antibiotic profile as shown in Table 5 depicts multiple antibiotic resistance (MAR) with multiple antibiotic resistant index (MARI) ranging from 0.36–0.5 in MAR-*V. cholerae* strains. This is also reflected in Table 4 as some members of the *V. cholerae* were resistant to carbapenem antibiotic members which are last choice antibiotics. Table 6 shows that 14/61 (23.0%) of the isolated *V. cholerae* were producing into the medium carbapenemase which resulted resistance to some members of the carbapenem antibiotics (ertapenem, doripenem). This is of high clinical concern as its distribution pose threat to possible outbreak, control/management failure, surveillance need and epidemiological investigations. Figure 2 shows the distribution of the isolates that produce carbapenemase and/or NDM-1 phenotype into the culture medium (Supplementary Figures SI 11–SI 13) as there was observation of an enlarged clear inhibition zone due to addition of EDTA impregnated disc while genotype was revealed in PCR gene detection of NDM-1 resistant genes was also shown in Table 7.

#### 3.3.4. Antibiotic Resistance Phenotypes

Amongst the various confirmed *V. cholerae* isolates examined, amikacin, meropenem, and gentamicin recorded 100% antibiotic susceptibility. Streptomycin, norfloxacin and imipenem activity did not produce zone diameter of inhibition to the level of susceptibility but were at the intermediate level following the EUCAST/CLSI [7,38] interpretation guidelines as shown in the Table 4 above. According to EUCAST [38], resistance, intermediate and sensitive interpretation were accessed separately as shown in Table 3 and Table 4. The highest level of resistance was observed amongst 47 (77.1%) isolates to cephalexin, while 26 (42.6%) isolates had more than ten resistant markers/phenotypes. These isolates were defined as multiple antibiotic resistant isolates in addition to other detected pathogens (see details in Table 3) as previously affirmed by Magiorakos et al. [56]. According to the study of Magiorakus and his colleagues, when an organism is resistant to more than three different class of antibiotics it is said to be a multiple antibiotic resistant (MDR) isolate [56]. Other details of resistant phenotypes are described in Figure 2 and Table 4, Table 5 and Table 6.

## 4. Discussion

Since the discovery of pathogens implication in multifaceted diseases of man and the environment, antibacterial susceptibility testing (AST) has been an invitro applicable/determinative step towards the control and management of disease borne pathogens. The previous discovery of (Alexander Fleming in 1920) penicillin [57] as well as its antecedents as antibacterial agent against pathogens has also affirmed such acclamation. According to EUCAST/CLSI [7,37,38,53] antibacterial susceptibility testing is basically necessary when a pathogen is of high clinical and epidemiological relevance with propensity to acquire resistance.

**Figure 3:**
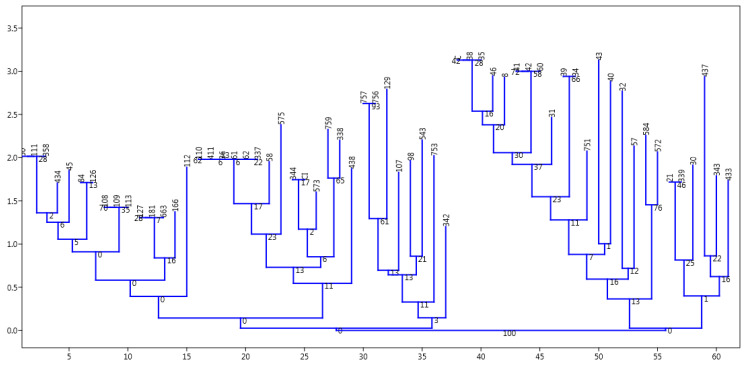
The above represent a dendrogram produced by the past3. software 3.14 Version when it was clustered using the neighbor joining clustering package and a Euclidean similarity index. It indicates that the isolates have evolved in five separate groups based on their susceptibility profile and a root of final branching.

**Figure 4:**
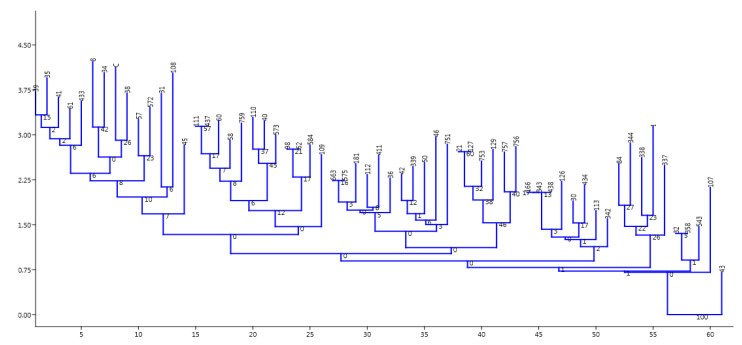
The above represent a dendrogram produced by the past3. software 3.14 Version when other sets of antibiotics used during experimental analysis clustered using the neighbor joining clustering package and a Euclidean similarity index. It indicates that the isolates have evolved arising from isolate 43 producing multiple clustered groups based on their susceptibility profile and a root of final branching.

Samples collected during this study confirmed sixty-one (8.0%) positive *V. cholerae* using molecular techniques out of 759 presumptive isolates indicating the occurrence of the potential pathogen in the sampled water (Table 5 and Supplementary Figure SI 2). The report is similar to the observation of Temba et al. [58] who affirmed the occurrence of *V. cholerae* in the estuaries of Tanzanian environment. All isolates were further sero-grouped and sero-typed as somatic non-agglutinating *V. cholerae* strains. Observing such potential cholera/acute watery diarrhea pathogens amongst environmental water sources necessitates further study on AST. It was observed that the various potential pathogens possess three to many antibiotic resistant phenotypes and markers as shown in the Table 3 and Section 3.3. This is an indication that present in the environmental estuaries and domestic water sources are multiple antibiotic resistant potential pathogens which pose threat to human and animal subjects that source the water for everyday domestic activities. This also reflects the earlier proposition of Manaia et al. [5] that the environmental water bodies are incubating black box for resistance amongst potential pathogens which inhabit estuaries. The study of Sulca et al. [59] and Uppal et al. [60] reported resistance to several antibiotics which is similar to the observations in this study. The occurrence of such multiple resistance markers/phenotypes observed during the study showed high multiple antibiotic resistant index (MARI) of about 0.5 for isolates that had sixteen multiple antibiotic resistant phenotype/markers. In addition, the isolates resistant profile also showed resistance to Carbapenems which are known last line of antibiotic choice for the management of infections and outbreaks amongst difficult to threat *V. cholerae*. The resistance profile summary is as follows: ertapenem 5 (8.2%), doripenem 1 (1.6%), chloramphenicol 39 (63.9%), tetracycline 43 (70.5%), doxycycline 45 (73.8%), azithromycin 19 (31.2%), ampicillin 34 (55.7%), augmentin 35 (57.4%), sulbactam-ampicillin 19 (31.2%), trimethoprime-sulfamethoxazole 40 (65.6%) cefuroxime 7 (11.5%). A higher number and percentage resistance was observed for nitrofurantoin 43 (70.5%), cephalexin 47 (77.1%), erythromycin 46 (75.4%), cephalothin 36 (59.0%), with few resistance occurring in ciprofloxacin 3 (4.9), nalidixic acid 7 (11.5%), while 100% susceptibility was observed amongst imipenem, amikacin, meropenem, norfloxacin, streptomycin and gentamicin (Figure 1a,b). This is similar to the report of Sulca et al. [59] who reported both sensitive and resistance to these groups of antibiotics but for a report which is at variance to our finding on amikacin. Amikacin was observed to have inhibited the entire tested organism with a zone appreciable with sensitivity as compared with the EUCAST/CLSI [38,53] guidelines during our study, which is contrary to the 14.7% resistance reported for amikacin by Sulca et al. [59]. Dengo-baloi et al. [61] reported a 13% resistance to azithromycin and a 100% resistance to nalidixic acid which is at variance with this study. Resistance to azithromycin and other fluoroquinolone antibiotics are also reported by other investigators [33]. The study of Ceccarelli et al. [62] also reported 8.2% resistance to streptomycin, while Wang et al. [63] reported resistance to gentamicin which was also at variance with what was observed in this current study. These variance reports might be associated with the region or environmental activities in their individual isolation sites or the flexible and changing nature of *V. cholerae* in diverse environment [64]. In the reports of Guevara et al. [65], resistance was not observed amongst the *Vibrio* strains but the trend changed over time as subsequent simultaneous report between 1992 and 2017 shows that [59,65,66,67,68,69,70,71,72] *V. cholerae* is emerging in resistant profile and multiple antibiotic resistance which have also extended from penicillanases or β-lactamases of various class to carbapenemases as affirmed during their study. This is further affirmed in this study as resistance was reported for extended spectrum β-lactamase and other novel carbapenemase resistance. Resistance to most commonly employed and important antibiotics are also another resistant nature observed amongst the *V. cholerae* isolated in this study. These antibiotics include ampicillin, azithromycin chloramphenicol, trimethoprime-sulfamethoxazole, tetracycline and doxycycline. This observation of high epidemiological relevance has aroused the need for a continuous surveillance/monitoring of the water bodies since it may serve as hot spot for spread of diseases. This also corroborated observations in the previous studies conducted by various *V. cholerae* investigators [61,62,63,73,74,75,76] on environmental strains. This study also reported *V. cholerae* multiple antibiotic resistances to some members of the cephalosporin groups of antibiotics. It was observed that 47 (77.1%) members of the cholera potential pathogen isolated during the study were resistant to cephalexin which is an oral cephem presumed to be applicable in the control and therapeutic management of a disease case. Other cephalosporin resistances observed are to ceftriaxone 3/61 (4.9%), cefazolin 22/61 (36.1%), cefuroxime 7/61 (11.5%), cefotaxime 7/61 (11.5%), cephalothin 36/61 (59.0%) and so on. Although from the EUCAST and CLSI documents, the cephalosporin members of antibiotics are only recommended for enterobacteriaceae (other members of the *Vibrio* family) such as *V. parahaemolyticus, V. mimicus, V. fluvialis* etc, but not recommended specifically for *V. cholerae* members. The observation of resistance amongst the *V. cholerae* to these antibiotic group is a potential determinant of epidemiological relevance especially when considering future strategies for management of cholera cases. Similar to the above observation is the reports from other clinical/environmental studies in the Asian continent (China, Mumbai, North India) (outside Africa) and other regions (Haiti) which have also reported similar occurrence of resistance to the cephalosporin group of antibiotics [59,76,77,78]. Their studies accessed water and stool samples and observed both O1/O139 and non-O1/nonO139 *V. cholerae* strains of diverse virulent and multiple antibiotic resistant determinants. The multiple antibiotic resistant natures and the subsequent resistance to cephalosporin, carbapenem and other antibiotic members is also indicative of drug pressure and new antibiotic resistance determinants in the isolates as 42.6% (26/61) were extended spectrum β-lactamase (ESβL) producers, 34.43% AmpC positive and 22.95% were producers of carbapenemase resistance. The molecular genotype of these resistant isolates reveal thus; ESβL (*blaTEM*; 25/29 (86.2%), *AmpC*; 17/30 (56.7%)), phenicols: (*catII* 14.39 (35.9%), *Flor* 18/39 (46.2%)), aminoglycosides: (*strA* (6.6%)), carbapenems; (*NDM-1*; 8/19 (42.1%) IMI (5.3%)), fluoroquinolones; (*qnrVC* 3/11 (27.3%), *QEP* 1/11 (9.1%)), cyclines (TetA 21/45 (46.7%)), folate pathway inhibitor: *TMP* 13/40 (32.5), *Sul2* 29/40 (72.5) as shown in Table 7 and Supplementary Figures SI 3–SI 7. The environmental non-O1/O139 *V. cholerae* were shown to produce into the medium biomolecules/enzyme which inhibited the antimicrobial effect of the tested antibiotics indicating antibacterial resistant genes amongst these bacteria. This corroborate the report of Wei et al. [79] that *NDM-1* resistance is reported amongst pathogens which are not attributable only to transfer of genes amongst unrelated potential pathogens but also include human factors e.g., personal hygiene practice, inter country travel and sanitation. It is important to note that these aforementioned human factors are the driving force for the spread of cholera as indicated by WHO, NICD and COVIS. One other deduction from the study was that amongst the examined isolates, the ones with multiple antibiotic resistance phenotype and those that produce new antibiotic resistant dynamics/genotypes are mainly from the river water and the wastewater treatment plant/receiving water bodies (Figure 2, Table 6). This indicates that the risk associated with contact with the water in these area of study is quiet enormous as it is imperative for individuals in these sub-urban region who use these water sources for domestic activities and recreational activities to desist from such application. A corroborated report was also documented from the study of Guo et al. [80] who reported an undesirable behavior of antibiotic resistance genotypes and/or phenotypes amongst isolates within wastewater treatment systems. Analyzing the various resistant profile using past3.zip software 3.14 as depicted in the Figure 3 and Figure 4, the distribution of resistant markers to commonly used antibiotic is shown in two major dendogram. It shows five clade clusters indicating the dissimilarity of the potential pathogens. Whereas, with the other non-common antibiotic profile, two clades were derived as isolate 43 belong to itself while other eight sub-clades were derived as observed in the dendogram of Figure 4 below. This could be inferred that as more effluent are released and antibiotic are applied for the management of infections associated with *V. cholerae*, there is an endless emergence of other resistant genotypes/phenotypes of clinical and epidemiological relevance. This study was able to affirm that AST and multiple antibiotic resistance gene determination possess astute relevance in epidemiological surveillance and steps towards infection control of *V. cholerae* especially those sourced from the environment. Reports from such resistant gene typing would also provide foundation for regulating antibiotic usage and public decision making.

## 5. Conclusions

This study describes the need for antibiotic susceptibility testing (AST) in continuous surveillance and monitoring strategy as a basis for epidemiological surveillance and steps towards control of the cholera. The potential environmental pathogen (non-O1/non-O139 *V. cholerae*) has been reported to be implicated in several disease cases of both children and adults in endemic and non-endemic area. Coastal water and the aquatic environment which has been the habitat of the potential pathogen had been observed to be an incubating spot of the environment for resistance sharing/transfer and continuous monitoring should be initiated to abort any emerging mechanism of resistance which the potential pathogens are today acquiring. Efforts towards controlling the indiscriminate use of antibiotics and unwanted release of effluent into the environment must also be checked regularly. This is urgent since the potential pathogen always spreads by contact with unhealthy water sources. Applying AST and resistant gene profiles would encourage policy making and appropriate regulation of chemical treatment of wastewater, antibiotic usage and release of antibiotic wastewater. The need to also increase the spectrum of antibiotics or antimicrobial agents for the management/control of acute watery diarrhea (AWD) and cholera infections is also reiterated as it is observed today that apart from the cholera pathogen, the environmental strains (environmental non O1/O139 *V. cholerae*) are implicated in disease cases and emerging in resistant mechanism. The observation of new antibiotic resistant phenotypes such as carbapenem resistant *V. cholerae (CRV.c),* (New Delhi Metallo β-lactamase producing *V. cholerae* (*NDM-1-V.c*)), extended spectrum β-lactamases producing *V. cholerae* (*ESβL-V.c*), other resistant phenotypes and their emerging resistant genotypes has opened another area of research and the need for development of better antimicrobial agents or alternative therapeutics in other to combat any future cholera outbreak in various locality. Since estuaries and water environment is a medium of *V. cholerae* spread, the concerted/continuous assessment of antibiotic profile of environmental strains remains a sure surveillance and monitoring strategy for emerging resistant pathogens. In addition, it will also assist control authorities in formulating policy, regulatory decisions, planning and implementation of disease control programmes.

## Figures and Tables

**Table 1:** Specific Primer Pairs and Annealing Temperature for *V. cholerae* and antibiotic resistant genes.

Target Gene	Primer Name	Sequence 5′–3′	Expected Band Size	Annealing Temp	Reference
16S rRNA	VF169	GGA TAA CC/TA TTG GAA ACG ATG	617 bp	53 °C	[45]
	VR744	CAT CTG AGT GTC AGT G/ATC TG			
OmpW	*V. choleF*	CACCAAGAAGGTGACTTTATTGTG	304 bp	64 °C	[50]
	*V. choler*	GGTTTGTCGAATTAGCTTCACC			
Vc Serogrp	*Vc-O1F*	GTTTCACTGAACAGATGGG	192 bp	55 °C	[19]
	*Vc-O1R*	GGTCATCTGTAAGTACAAC			
	*Vc-O139F*	AGCCTCTTTATTACGGGTGG	449 bp	55 °C	[19]
	*Vc-O139R*	GTCAAACCCGATCGTAAAGG			
*TetA*	*TetA-F*	GTAATTCTGAGCACTGTCGC	950 bp	55 °C	[34]
	*TetA-R*	CTGCCTGGACAACATTGCTT			
*IntI*	*intI-F*	GCTGGATAGGTTAAGGGCGG	521 bp	55 °C	[43]
	*intI-R*	CTCTATGGGCACTGTCCACATTG			
*FLOR*	*flor F*	TTATCTCCCTGTCGTTCCAGCG	586 bp	55 °C	[43]
	*flor R*	CCTATGAGCACACGGGGAGC			
*Sul*	*sul2 F*	AGGGGGCAGATGTGATCGC	625 bp	58 °C	[43]
	*sul2 R*	TGTGCGGATGAAGTCAGCTCC			
*TMP*	*TMP-F*	TGGGTAAGACACTCGTCATGGG	389 bp	60.5 °C	[43]
	*TMP-R*	ACTGCCGTTTTCGATAATGTGG			
QNRVC	*qnrVC-F*	CCCTCGAGCATGGATAAAACAGACCAGTTATA	521 bp	62 °C	[6]
	qnrVC-R	CGGGATCCTTAGTCAGGAACTACTATTAAACCT			
QEP	*qepA-F*	AACTGCTTGAGCCCGTAGAT	596 bp	59 °C	[6]
	*qepA-R*	GTCTACGCCATGGACCTCAC			
AMPC	*ampC-F*	TTCTATCAAACTGGCARCC	545 bp	45 °C	[31,34]
	*ampC-R*	CCYTTTTATGTACCCAYGA			
NDM	*blaNDM-1-F*	GGTTTGGCGATCTGGTTTTC	621 bp	52 °C	[51]
	*blaNDM-1-R*	CGGAATGGCTCATCACGATC			
FQ	*FQ-1-F*	ATGACGCCATTACTGTATAA	566 bp	54 °C	[6]
	*FQ-1-R*	GATCGCAATGTGTGAAGTTT			
QP	*QP-1-F*	GATAAAGTTTTTCAGCAAGAGG	657 bp	55 °C	[6]
	*QP-2-R*	ATCCAGATCGGCAAAGGTTA			
Cat	*catII-F*	ACACTTTGCCCTTTATCGTC	542 bp	50 °C	[34]
	*catII-R*	TGAAAGCCATCACATACTGC			
STR	*str-F*	CTTGGTGATAACGGCAATTC	348 bp	50 °C	[34]
	*str-R*	CCAATCGCAGATAGAAGGC			
	*aadA-F*	GTGGATGGCGGCCTGAAGCC	525 bp	50 °C	[34]
	*aadA-R*	AATGCCCAGTCGGCAGCG			
VIM	*VIM-F*	GATGGTGTTTGGTCGCATA	390 bp	55 °C	[33]
	*VIM-R*	CGAATGCGCAGCACCAG			
GES	*GES-F*	AGTCGGCTAGACCGGAAAG	399 bp	57 °C	[33]
	*GES-R*	TTTGTCCGTGCTCAGGAT			
IMP	*imp-F*	TTGACACTCCATTTACDG	139 bp	55 °C	[33]
	*imp-R*	GATYGAGAATTAAGCCACYCT			
TEM	*blaTEM-F*	ATCAGCAATAAACCAGC	515 bp	56 °C	[34,51]
	*blaTEM-R*	CCCCGAAGAACGTTTTC			
SHV	*blaSHV-F*	AGGATTGACTGCCTTTTTG	390 bp	55 °C	[34,51]
	*blaSHV-R*	ATTTGCTGATTTCGCTCG			

**Table 2:** The total number of presumptive isolates and confirmed *V. cholerae*, their occurrence and their site within six months.

Location Code	Sampled Water Type	March	PCR Confirmed	April	PCR Confirmed	May	PCR Confirmed	June	PCR Confirmed	July	PCR Confirmed	August	PCR Confirmed	Total *V. Cholerae* CONFIRMED
Sample H	Irrigation Canal	Nil	Nil	15	0	Nil	Nil	8	0	6	0	5	0	
	Dam	Nil	Nil	13	1	Nil	Nil	10	0	5	0	6	1	
	River	Nil	Nil	29	1	Nil	Nil	11	0	8	0	9	0	
	WWTP	Nil	Nil	Nil	Nil	Nil	Nil	Nil	Nil	Nil	Nil	Nil	Nil	
	Total prespt/conf	Nil	Nil	57	2	Nil	Nil	29	0	19	0	20	1	3
Sample C	Irrigation Canal	11	0	8	0	7	0	6	0	5	0	7	0	
	Dam	19	2	11	0	9	0	6	0	6	0	7	1	
	River	18	1	17	0	11	0	7	0	8	0	8	1	
	WWTP	3	0	4	0	2	0	3	0	2	0	3	0	
	Total prespt/conf	51	3	40	0	29	0	22	0	21	0	25	2	5
Sample Q	Irrigation Canal	15	2	10	1	8	0	6	0	6	1	7	1	
	Dam	13	2	12	1	9	0	7	0	11	1	7	1	
	River	16	3	14	1	11	1	9	1	14	2	9	2	
	WWTP	23	6	19	2	14	1	11	1	17	2	11	2	
	Total prespt/conf	67	13	55	5	42	2	33	2	48	6	34	6	34
Sample CF	Irrigation Canal	7	2	9	1	5	0	5	0	4	0	4	0	
	Dam	9	1	8	1	5	0	6	0	5	0	5	1	
	River	11	2	11	1	7	1	9	1	5	1	6	1	
	WWTP	15	2	11	1	6	1	Nil		7	1	7	1	
	Total prespt/Conf	42	7	39	4	23	2	20	1	21	2	22	3	19

**Table 3:** Difference in microbial (*V.cholerae*) count (10^−2^) among 3 plants, using ANOVA.

PLANTS	*N*	Mean of Count ± S.E.M	F	P
cof WWTP	18	18.17 ± 8.23	26.78	0.00 *
QT WWTP	18	244.61 ± 44.86		
Cath WWTP	18	0.00 ± 0.00		
Total	54	87.59 ± 21.36		

* Significance: *p* < 0.05. The result presented in the table shows that there is a significant difference among: cofWWTP (Mean = 18.17, SEM = 8.23); QTWWTP (Mean = 244.61, SEM = 44.86) and CathWWTP (Mean = 0.00, SEM = 0.00), (*p* < 0.05). the mean values obtained showed that, the microbial count was higher at QTWWTP.

**Table 4:** Antibiotic susceptibility profile of the various *V. cholerae* Isolates.

Antibiotic Class/Group	Antibiotic Types	Sensitive (%)	*V. cholerae* (*N* = 61)	Resistance (%)
			Intermediate (%)	
Penicillin	AP-25 µg	16 (26.2)	10 (16.4)	34 (55.7)
β -Lactam/β-Lactamase Inhibitor	AUG-30 µg	25 (41.0)	1 (1.6)	35 (57.4
	SAM-20 µg	31 (50.8)	11 (18.0)	19 (31.2)
	PTZ-110 µg	59 (96.7)	0	2 (3.3)
Cephalosporin/Cephem	CRO-30 µg	57 (93.4)	1 (1.6)	3 (4.9)
	CPM-30 µg	57 (93.4)	3 (4.9)	1 (1.6)
	CXM-30 µg	52 (85.3)	2 (3.3)	7 (11.5)
	CAZ-30 µg	59 (96.7)	0	2 (3.3)
	CZ-30 µg	33 (54.1)	6 (9.8)	22 (36.1)
	CTX-30 µg	54 (88.5)	0	7 (11.5)
	CFX-30 µg	7 (11.5)	7 (11.5)	47 (77.1)
	KF-30 µg	11 (18.0)	14 (23.0)	36 (59.0)
Carbapenems	IMI-10 µg	59 (96.7)	2 (3.3)	0
	MEM-10 µg	61 (100)	0	0
	ETP-10 µg	53 (86.9)	3 (4.9)	5 (8.2)
	DOR-10 µg	59 (96.7)	1 (1.6)	1 (1.6)
Macrolides	ATH-15 µg	42 (68.9)	0	19 (31.2)
	E-15 µg	15 (24.6)	0	46 (75.4)
Phenicols	C-30 µg	18 (29.5)	4 (6.6)	39 (63.9)
Aminoglycosides	GM-10 µg	61 (100)	0	0
	S-300 µg	60 (98.4)	1 (1.6)	0
	AK-30 µg	61 (100)	0	0
	K-30 µg	42 (68.9)	3 (4.9)	16 (26.2)
Fluoroquinolones	CIP-5 µg	50 (81.9)	8 (13.1)	3 (4.9)
	LEV-5 µg	59 (96.7)	1 (1.6)	1 (1.6)
	NOR-10 µg	56 (91.8)	5 (8.2)	0
	NA-30 µg	44 (72.1)	10 (16.4)	7 (11.5)
Tetracycline	T-30 µg	15 (24.6)	3 (4.9)	43 (70.5)
	DXT-30 µg	15 (24.6	1 (1.6)	45 (73.8)
Folate Pathway Inhibitor	TS-25 µg	20 (32.8)	1 (1.6)	40 (65.6)
Nitrofuran	NI-200 µg	16 (26.2)	2 (3.3)	43 (70.5)

(Ceftazidime (CAZ-30 µg), Cefepime (CPM-30 µg), Cefotaxime (CTX-30 µg), Ceftriaxone (CRO-30 µg), Cefuroxime (CXM-30 µg), Cephalexin (CFX-30 µg), Cephalothin (KF-30 µg), Cefazolin (CZ-30 µg), Nitrofurantoin (NI-200 μg), Chloramphenicol (C-30 μg), Trimethoprime-Sulfamethoxazole (TS-25 μg), Ampicillin (AP-10 μg), Amoxicillin-Clavulanate (AUG-30 μg), Piperacillin-Tazobactam (PTZ-110 μg), Ampicillin-Sulbactam (SAM-20 μg), Gentamicin (Gm-30 μg), Amikacin (AK-30 μg), Streptomycin (S-30 μg), Kanamycin (K-30 μg), Imipenem (Imi-30 μg), Ertapenem (ETP-10 μg), Meropenem (Mem-10 μg), Doripenem (Dor-10 μg), Tetracycline (T-30 μg), Doxycycline (DXT-30 μg), Erythromycin (E-15 µg), Azithromycin (ATH-15 μg), Ciprofloxacin (CIP-5 µg), Levofloxacin (Lev-5 µg), Nalidixic acid (NA-30 µg), Norfloxacin (Nor-10 µg) and Polymyxin B (PB-300 µg)).

**Table 5:** Multiple antibiotic resistant index and resistant markers/phenotypes of *V. cholerae* Isolates.

Isolates	Resistant Markers/Phenotypes of Isolates	NO. R	NO. I	S.NO	MARI
21	AP, Ni, DXT, T, TS, AUG, SAM, CTX, KF, E, CFX	11	0	18	0.344
50	C, NI, DXT, T, TS, AUG, SAM, KF, E, CFX, NA	9	2	18	0.281
84	AP, C, NI, DXT, T, TS, AUG, SAM, KF, E, CFX, NA	12	2	15	0.375
98	CXM, AP, CZ, C, NI, DXT, T, KF, E, CFX	10	1	18	0.313
107	CXM, AP, CZ, C, NI, DXT, T, TS, PTZ, AUG, SAM, KF, E, CFX	14	0	15	0.438
108	AP, C, NI, DXT, T, TS, PTZ, SAM, CFX, NA	10	2	17	0.313
109	AP, C, NI, DXT, T, TS, SAM, E, CFX	9	3	17	0.281
110	AP, K, C, NI, DXT, T, TS, E, ATH	9	1	19	0.281
112	CZ, DOR, C, NI, DXT, T, TS, AUG, KF, E, CFX, ATH	12	4	13	0.375
166	AP, CZ, C, NI, DXT, T, TS, AUG, KF, E, CFX	11	1	17	0.344
129	CXM, CAZ, CZ, ETP, C, NI, DXT, T, TS, AUG, CTX, KF, CFX	14	6	10	0.438
127	AP, CZ, C, NI, DXT, T, TS, AUG, SAM, CTX, KF, E, CFX	13	0	16	0.406
126	AP, C, NI, DXT, T, TS, AUG, E, CFX	9	2	18	0.281
113	AP, C, NI, DXT, T, TS, AUG, E, CFX	9	3	17	0.281
181	AP, CZ, C, NI, DXT, T, TS, AUG, KF, E, CFX, ATH	12	0	17	0.375
339	AP, NI, DXT, T, TS, AUG, SAM, KF, E, CFX, ATH	11	1	17	0.344
411	AP, K, C, NI, DXT, T, TS, AUG, E, CFX, ATH	11	2	16	0.344
438	AP, CZ, K, C, NI, DXT, T, AUG, KF, E, CFX	11	0	18	0.344
358	C, NI, DXT, T, TS, AUG, SAM, KF, E, CFX	10	0	19	0.313
757	CRO, CXM, AP, CZ, ETP, C, NI, DXT, T, TS, AUG, CTX, KF, E, CFX, ATH	16	1	13	0.500
756	CRO, CXM, AP, CZ, ETP, C, NI, DXT, T, TS, AUG, CTX, KF, E, CFX, ATH	16	3	11	0.500
753	CZ, ETP, NI, DXT, AUG, CTX, KF, E, CFX	9	4	16	0.281
663	AP, CZ, C, NI, DXT, T, TS, AUG, KF, E, CFX, ATH	12	1	16	0.375
543	CXM, AP, CZ, ETP, C, NI, T, DXT, AUG, SAM, KF, E, CFX	13	2	14	0.406
36	AP, K, C, NI, DXT, T, TS, AUG, E, CFX, ATH	11	2	16	0.344
759	AP, CZ, K, C, NI, DXT, T, TS, E, CFX	9	3	18	0.281
573	AP, K, C, NI, DXT, T, TS, E, ATH	9	3	17	0.281
575	CXM, AP, K, C, NI, DXT, T, TS, AUG, KF, E, CFX, ATH	13	1	15	0.406
62	AP, K, C, NI, DXT, T, TS, KF, E, CFX	10	0	19	0.313
344	AP, CZ, K, C, NI, DXT, T, TS, AUG, SAM, KF, E, CFX, CIP, NA	15	1	13	0.469
338	AP, CZ, K, C, DXT, T, TS, AUG, SAM, KF, E, CFX, ATH, CIP	14	2	13	0.438
337	AP, K, C, NI, DXT, T, TS, AUG, SAM, KF, E, CFX, CIP	13	1	15	0.406
I	AP, CZ, K, C, NI, DXT, T, TS, AUG, SAM, KF, E, CFX, ATH, NA, LEV	16	2	11	0.500

NO. R represents numbers of resistant marker/phenotypes, NO. I represent numbers of markers at intermediate range while NO. S represents numbers with sensitive markers/phenotype for the tested antibiotics in each *V. cholerae* isolated. MARI is the multiple antibiotic resistant index of each isolate. (ceftazidime (CAZ-30 µg), cefepime (CPM-30 µg), cefotaxime (CTX-30 µg), ceftriaxone (CRO-30 µg), cefuroxime (CXM-30 µg), cephalexin (CFX-30 µg), cephalothin (KF-30 µg), cefazolin (CZ-30 µg), nitrofurantoin (NI-200 μg), chloramphenicol (C-30 μg), trimethoprime-sulfamethoxazole (TS-25 μg), ampicillin (AP-10 μg), amoxicillin-clavulanate (AUG-30 μg), piperacillin-tazobactam (PTZ-110 μg), ampicillin-sulbactam (SAM-20 μg), gentamicin (Gm-30 μg), amikacin (AK-30 μg), streptomycin (S-30 μg), kanamycin (K-30 μg), imipenem (Imi-30 μg), ertapenem (ETP-10 μg), meropenem (Mem-10 μg), doripenem (Dor-10 μg), tetracycline (T-30 μg), doxycycline (DXT-30 μg), erythromycin (E-15 µg), azithromycin (ATH-15 μg), ciprofloxacin (CIP-5 µg), levofloxacin (Lev-5 µg), nalidixic acid (NA-30 µg), norfloxacin (Nor-10 µg) and polymyxin B (PB-300 µg)).

**Table 6:** The occurrence of various resistant phenotypes by the tested *V. cholerae* isolates.

Resistant Phenotypes	Numbers of Accessed Isolates (%)	Numbers Showing Positive Phenotype (%)
AmpC	30 (49.2)	21 (34.4)
ESβL	29 (47.5)	26 (42.6)
NDM-1	19 (31.1)	14 (23.0)

The following describes the codes as NDM-1 depicts New Delhi Metalobetalactamase type 1, ESβL depicts Extended Spectrum betalactamase, while AmpC depicts a class C betalactamase gene.

**Table 7:** The Occurrence of various Resistant Genotypes by the test *V. cholerae* strains.

Antibiotics Groups	Total Number of Vibrio Cholerae (%)	Resistant Genes Determined	Number/Percentage Resistance Observed (%)
BetaLactam/β-lactamase inhibitors/Cephalosporins	29/61 (47.5%)	*blaTEM*	25/29 (86.2)
		*blaSHV*	2/29 (6.9)
		*blaCTXM*	Nil
	30/61 (49.2%)	*AmpC*	17/30 (56.7)
Carbapenems	19/61 (31.1%)	*NDM-1*	8/19 (42.1)
		*GES*	Nil
		*IMP*	1/19 (5.3)
		*VIM*	Nil
Phenicols	(39/61, 63.9%)	*Flor*	18/39 (46.2)
		*CatII*	14/39 (35.9)
Fluoroquinolones	11/61 (18.33%)	*QP1*	Nil
		*FQ*	Nil
		*QNRVC*	3/11 (27.3)
		*QEP*	1/11 (9.1)
Aminoglycosides	61/61 (100)	*strA*	4/61 (6.6)
		*aadA*	Nil
Folate Pathway Inhibitor/Trimetoprime-Sulphametoxazol	40/61, (65.6%)	*TMP*	13/40 (32.5)
		*Sul2* *INT1*	29/40 (72.5) 26/40 (65.0)
Cyclines	45/61 (73.8%)	*tetA*	21/45 (46.7)

The following describes the resistant genotypes of the various groups of antibiotic and the percentage detected as NDM-1, ESβL, AmpC and other resistant genes.

## Supplementary Materials

The following are available online at https://www.mdpi.com/1660-4601/17/16/5685/s1, Figure SI 1; Gel photo of the Genus specific *16SrRNA gene* detection; Figure SI 2; Photomicrogram of *OmpW* gene detection; Figure SI 3; Photomicrogram of *blaTEM* resistant gene detection; Figure SI 4; Photomicrogram of *tetA* resistant gene detection; Figure SI 5; Photomicrogram of *Flor* resistant gene detection; Figure SI 6; Photomicrogram of a rear integrase; Figure SI 7; Photomicrogram of a chloramphenicol (*catII*) resistant gene detection; Figure SI 8; Photomicrogram of positive AmpC phenotype amongst isolates; Figure SI 9; Photomicrogram of positive ESβLs phenotype amongst isolates; Figure SI 10; Photomicrogram of positive ESβLs phenotype amongst isolates; Figure SI 11; Photomicrogram of positive NDM phenotype amongst isolates; Figure SI 12; Photomicrogram of positive NDM phenotype amongst isolates; Figure SI 13; Photomicrogram of positive NDM phenotype amongst isolates.
