# Supplementary material for: Antibiotic Susceptibility Testing (AST) Reports: A Basis for Environmental/Epidemiological Surveillance and Infection Control Amongst Environmental Vibrio cholerae
Source: Int J Environ Res Public Health. 2020 Aug 6;17(16):5685. doi: 10.3390/ijerph17165685 (PMC7460427; doi:10.3390/ijerph17165685)
Supplement: Supplementary file 1 [file ijerph-17-05685-s001.pdf]

Supplementary Figures SI 1 – SI 13

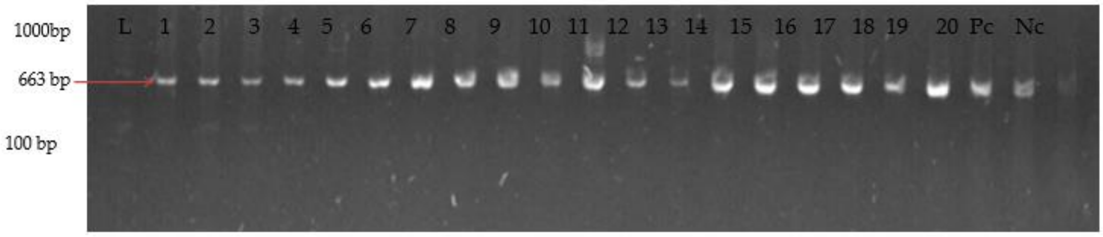

Figure SI 1. Gel photo of the Genus specific *16SrRNA gene* detection L represents a molecular marker of 1.2kb, Pc is a positive control *DSM 19130 V. cholerae*, Nc is a negative control *DSM 8224 Plesiomonas shigelloides*, while numbers 1-20 are positive isolates.

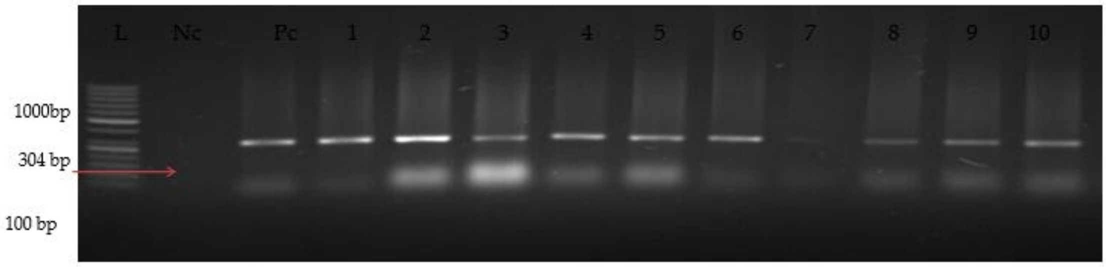

Figure SI 2. Photomicrogram of *OmpW* gene detection, L represents a molecular marker of 1.2kb, Pc is a positive control *DSM 19130 V. cholerae*, Nc is a negative control *DSM 8224 Plesiomonas shigelloides*, while numbers 1-10 are positive isolates.

Antibiotic Resistant and Resistance Associated Genes

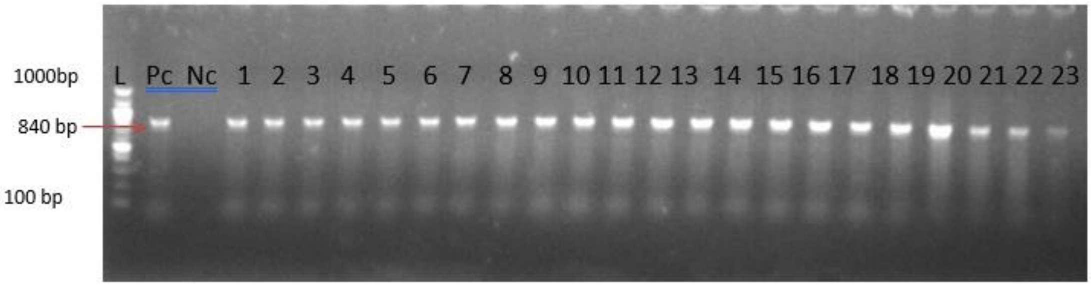

Figure SI 3. Photomicrogram of blaTEM resistant gene detection at 840 bp, L represents a molecular marker of 1.2kb, Pc is a positive control DSM 19130 *V. cholerae*, Nc is a negative control DSM 8224 *Plesiomonas shigelloides*, while numbers 1-23 are positive isolates.

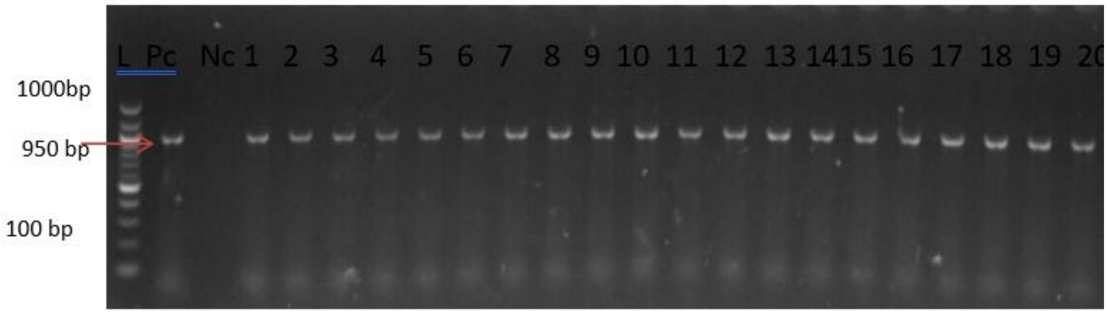

Figure SI 4. Photomicrogram of tetA resistant gene detection (950 bp), L represents a molecular marker of 1.2kb, Pc is a positive control DSM 19130 *V. cholerae*, Nc is a negative control DSM 8224 *Plesiomonas shigelloides*, while numbers 1-20 are positive isolates.

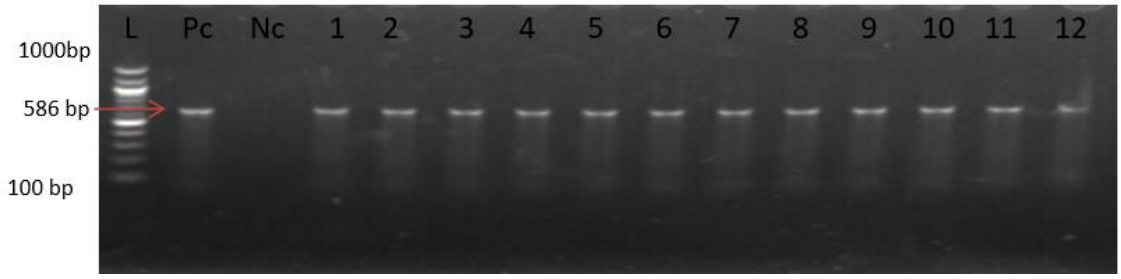

Figure SI 5. Photomicrogram of Flor resistant gene detection (586 bp), L represents a molecular marker of 1.2kb, Pc is a positive control DSM 19130 *V. cholerae*, Nc is a negative control DSM 8224 *Plesiomonas shigelloides*, while numbers 1-12 are positive isolates.

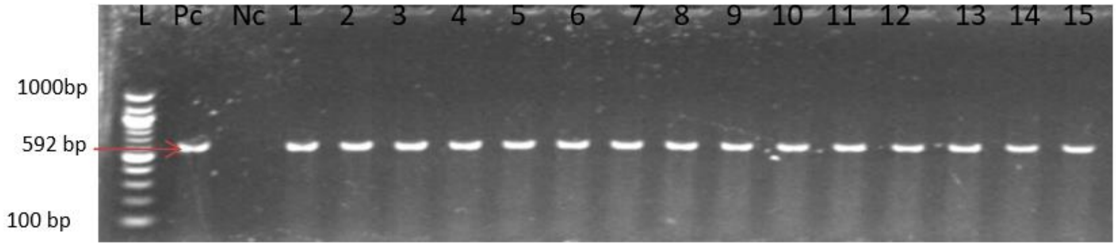

Figure SI 6. Photomicrogram of a rear integrase (INT1) gene detection (592 bp), L represents a molecular marker of 1.2kb, Pc is a positive control DSM 19130 *V. cholerae*, Nc is a negative control DSM 8224 *Plesiomonas shigelloides*, while numbers 1-15 are positive.

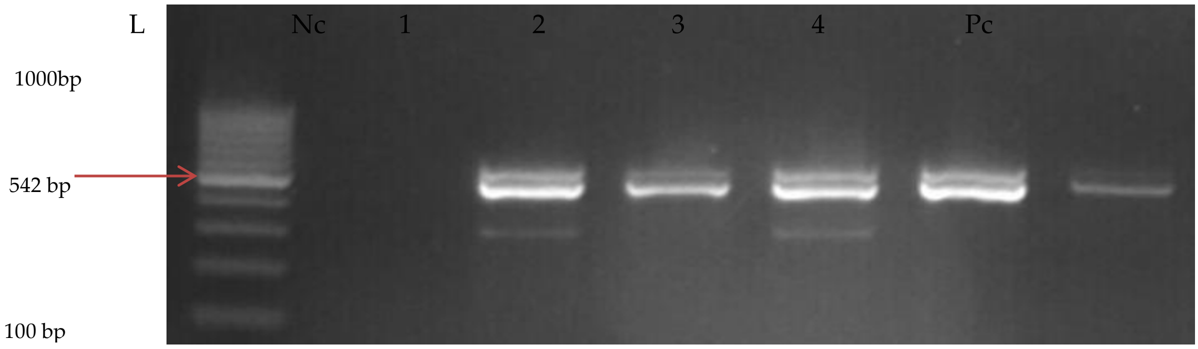

Figure SI 7. Photomicrogram of a chloramphenicol (catII) resistant gene detection (542 bp), L represents a molecular marker of 1.2kb, Pc is a positive control DSM 19130 *V. cholerae*, Nc is a negative control DSM 8224 *Plesiomonas shigelloides*, while numbers 1–4 are positive.

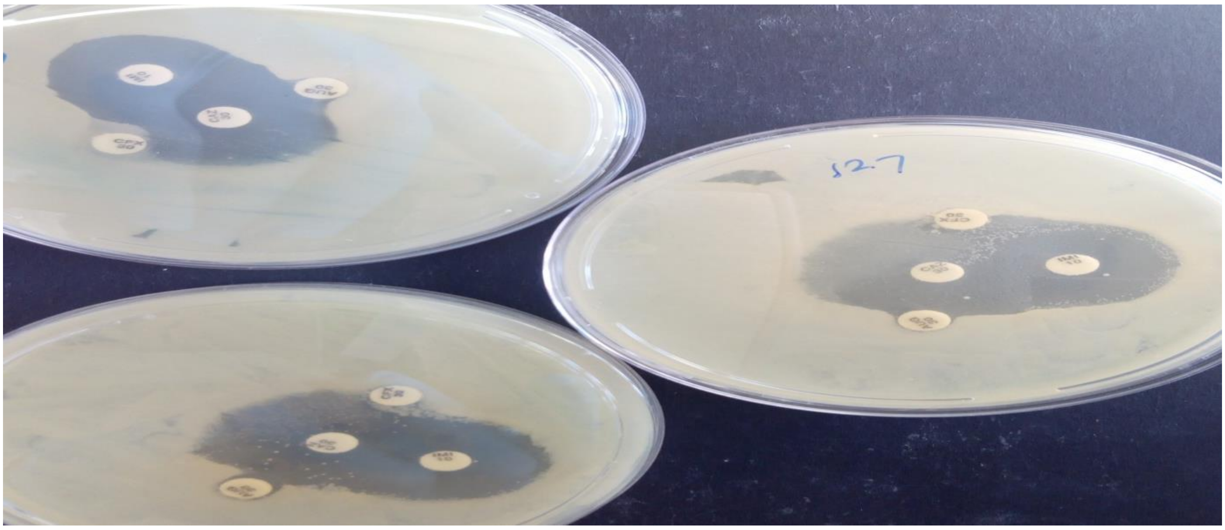

Figure SI 8. Photomicrogram of positive AmpC phenotype amongst isolates. An obvious blunting or flattening of the zone of inhibition between ceftazidime disk and other inducing antibiotics disks (imipenem, cefoxitin and amoxicillin-clavulanate)

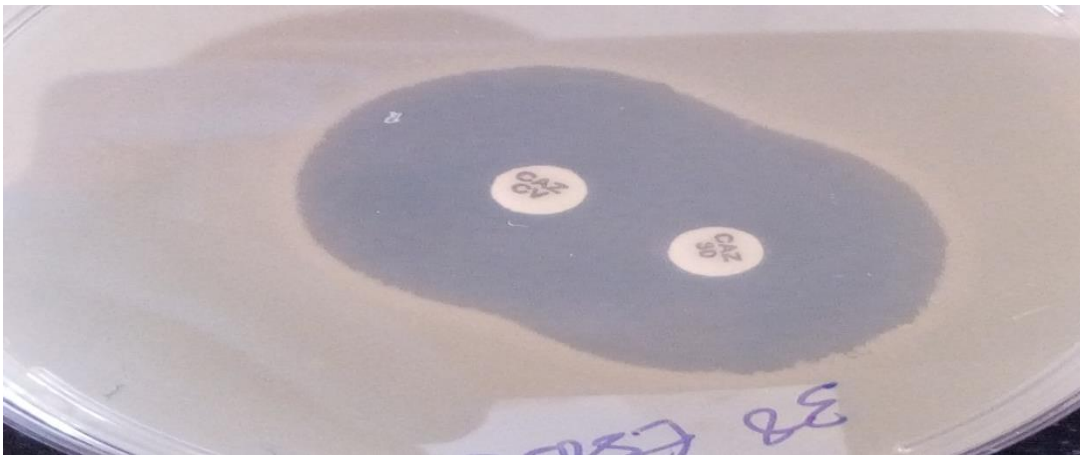

Figure SI 9. Photomicrogram of positive ESβLs phenotype amongst isolates. An observation of ≥ 5 mm increase in zone of inhibition diameter for Ceftazidime in the synergy test

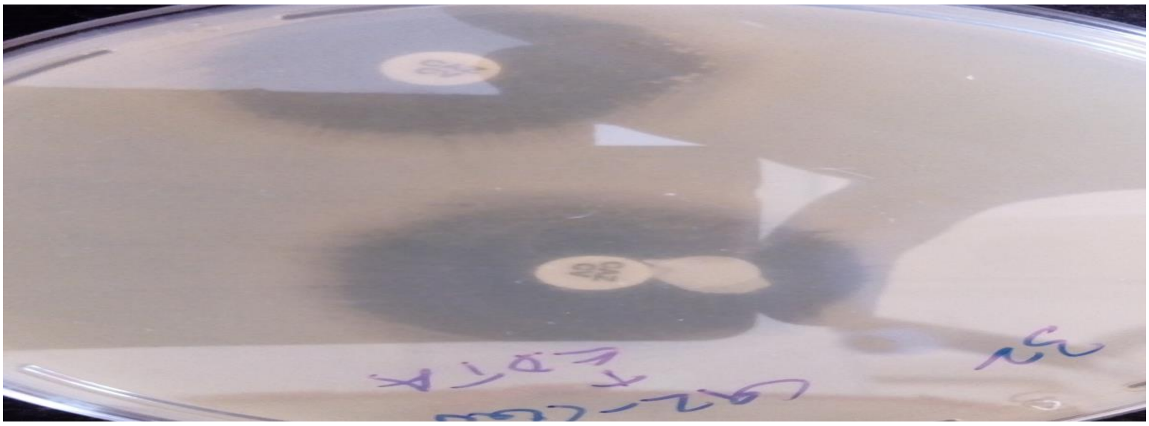

Figure SI 10. Photomicrogram of positive ESβLs phenotype amongst isolates. An observation of ≥ 5 mm increase in zone of inhibition diameter for Ceftazidime in the synergy test

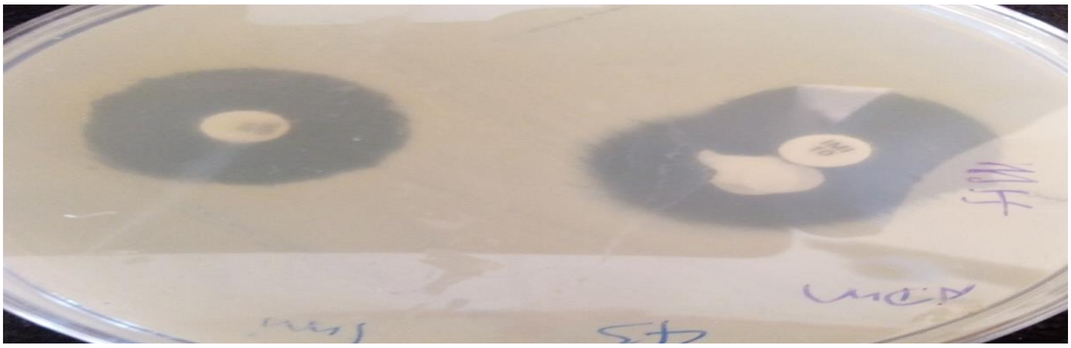

Figure SI 11. Photomicrogram of positive NDM phenotype amongst isolates. The observation of ≥4 mm zone of inhibition in the EDTA fortified disc is indicative of a carbapenemase producing isolate.

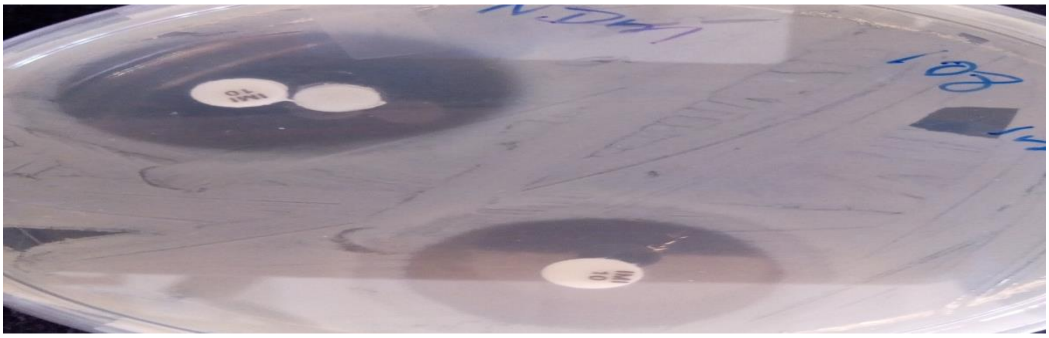

Figure SI 12. Photomicrogram of positive NDM phenotype amongst isolates. The observation of ≥4 mm zone of inhibition in the EDTA fortified disc is indicative of a carbapenemase producing isolate.

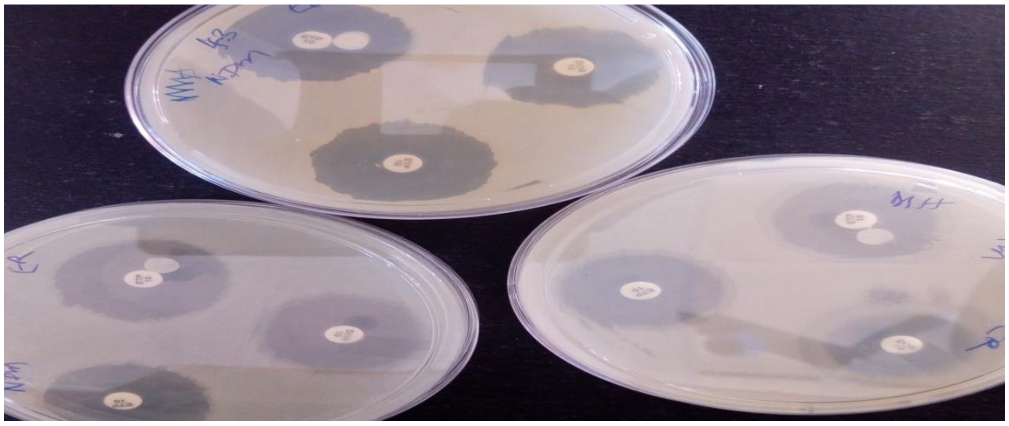

Figure SI 13. Photomicrogram of positive NDM phenotype amongst isolates. The observation of ≥4 mm zone of inhibition in the EDTA fortified disc is indicative of a carbapenemase producing isolate.
